
JOURNAL INFORMATION
==============================
NLM Title Abbreviation: ScientificWorldJournal
Journal ID: ScientificWorldJournal
NLM Journal ID: 101131163
Journal ID: TSWJ

The Scientific World Journal

ISSN: 2356-6140
EISSN: 1537-744X
Publisher: Wiley

ARTICLE INFORMATION
==============================
PMCID: PMC5848732
PMID: 19030757
DOI: 10.1100/tsw.2008.151
Article ID: 757810
Article version: 1
Subjects: Letter to the Editor

Reversibility of Cataracts in Diabetes

Sanz Silvia
Lillo Juan
Arruga Jorge *
Department of Ophthalmology, Hospital Universitari de Bellvitge, Feixa Llarga s/n. 08907 L'Hospitalet del Llobregat, Barcelona, Spain
*Jorge Arruga: jarruga@bellvitgehospital.catAcademic Editor: Daniel Batlle

Publication date: 2008
Electronic publication date: 2008 Nov 19
Volume: 8
First page: 1148
Last page: 1149
Received 2008 Nov 17; Accepted 2008 Nov 17
Copyright: Copyright © 2008 Silvia Sanz et al.
Copyright year: 2008
License: This is an open access article distributed under the Creative Commons Attribution License, which permits unrestricted use, distribution, and reproduction in any medium, provided the original work is properly cited.
License URL: https://creativecommons.org/licenses/by/3.0/

==============================
Letter to the Editor
*TheScientificWorldJOURNAL* (2008) 8, 1148–1149
ISSN 1537-744X; DOI 10.1100/tsw.2008.151

TheScientificWorldJOURNAL
www.thescientificworld.com

### Reversibility of Cataracts in Diabetes

Silvia Sanz, Juan Lillo, and Jorge Arruga*

*Department of Ophthalmology, Hospital Universitari de Bellvitge, Feixa Llarga s/n. 08907 L'Hospitalet del Llobregat, Barcelona, Spain*

**E-mail**: jarruga@bellvitgehospital.cat

*Received November 17, 2008; Accepted November 17, 2008; Published November 19, 2008*

---

Dear Editor,

The article by Ramkumar and Basti[1] describes the acute onset of a bilateral cataract related to the onset of diabetes, and the eventual reversal of the lens opacities after accomplishing a fall in blood glucose levels with insulin therapy. Reports of this ocular manifestation of diabetes, also called “acute sugar cataract”, are scarce[2].

The development of a reversible lens opacification in human diabetics is associated with changes in the lens hydration producing oedema, vacuole formation, and increased membrane permeability[3]. In addition, other mechanisms, such as oxidative stress, are involved.

As described by the authors[1], the typical morphology of these cataracts is a subcapsular opacity. This peculiar appearance resembling a rosette is due to the fact that the cortical sutures of the lens act as a backbone and, thus, opacities radiate from a central subcapsular plaque towards the periphery. Conversely, the resolution of the opacification follows a centripetal direction due to the repair or formation of new lens fibres by the equatorial epithelial cells[4].

The patient may complain of blurred vision as a consequence of oedema producing a disruption of the orderly arranged lens fibres and altering their transparency, and also because of transient changes in the lens refractive index[3]. In most of the cases, the symptoms are reversed once glucose levels are under control. However, if hyperglycaemia persists, alterations in cell membrane may produce electrolyte and biochemical changes that cause cell death and irreversible cataracts.

Different experimental studies help to explain the pathogenesis of the cataracts in these cases[5]. Under normal conditions, the main source of energy of the human lens is glucose, which is metabolised through the glycolytic pathway. When blood glucose levels are high in diabetic patients, the lens is exposed to a hyperosmotic aqueous humour and a progressive increase of its glucose concentration is produced. This fact causes a saturation of the glycolytic pathway and the activation of the sorbitol pathway, with an increase in aldose reductase activity. Glucose is then converted to sorbitol and fructose, and a depletion of nicotinamide adenine dinucleotide phosphate ensues.

$$\text{Glucose} + \text{NADPH} + \text{H}^+ \xrightarrow{\text{aldose reductase}} \text{sorbitol} + \text{NADP}^+$$

$$\text{Sorbitol} + \text{NAD}^+ \xrightarrow{\text{sorbitol deshydrogenase}} \text{fructose} + \text{NADH} + \text{H}^+$$

*Corresponding author.
©2008 with author.
Published by TheScientificWorld; www.thescientificworld.com

The accumulation of these substances within the lens fibres creates an osmotic hydration of the tissue, with a gradual swelling of the lens. When normal levels of glycaemia are restored, an excess of sorbitol persists in the lens, as it diffuses more slowly than glucose. Because of that sustained lens osmolality, water is still drawn into the lens, increasing the swelling. Jacob and Duncan[6] called this phenomenon "double osmotic shock", and this fact explains why refractive changes may persist some weeks after the restoration of glucose levels.

In summary, the case reported emphasises that in patients developing an acute cataract, a systemic evaluation should be done to rule out diabetes mellitus or other metabolic disorders. In addition, because of the potential reversibility of this type of cataract, in most instances surgical procedures will be unnecessary.

*Silvia Sanz, MD*
*Juan Lillo, MD*
*Jorge Arruga, MD*

### REFERENCES

1. Ramkumar, H. and Basti, S. (2008) Reversal bilateral rosette cataracts with glycemic control. *TheScientificWorldJOURNAL* **8,** 1150–1151.
2. Sharma, P. and Vasavada, A.R. (2001) Acute transient bilateral diabetic posterior subcapsular cataracts (1). *J. Cataract Refract. Surg*. **27,** 789–794.
3. Bron, A.J., Sparrow, J., Brown, A.P., et al. (1993) The lens in diabetes. *Eye* **7,** 260–275.
4. Kaufman, L.P. (2002) *Alm A. Adler´s Physiology of the Eye: Clinical Application*. 10th ed. Sect. 3: The Lens. Mosby, St. Louis.
5. Hedge, K.R., Henein, M.G., and Varma, S.D. (2003) Establishment of the mouse as a model animal for the study of diabetic cataracts. *Ophthalmic Res*. **35,** 12–18.
6. Jacob, T.J.C. and Duncan, G. (1982) Glucose-induced membrane permeability changes in the lens. *Exp. Eye Res*. **34,** 445–453.

**This article should be cited as follows:**

Sanz, S., Lillo, J., and Arruga, J. (2008) Reversal of bilateral rosette cataracts with glycemic control. *TheScientificWorldJOURNAL* **8**, 1148–1149. DOI 10.1100/tsw.2008.151.
